# Common and specific genomic sequences of avian and human extraintestinal pathogenic *Escherichia coli *as determined by genomic subtractive hybridization

**DOI:** 10.1186/1471-2180-7-81

**Published:** 2007-08-30

**Authors:** Subhashinie Kariyawasam, Jennifer A Scaccianoce, Lisa K Nolan

**Affiliations:** 1 Department of Veterinary Microbiology and Preventive Medicine, College of Veterinary Medicine, 1802 Elwood Drive, VMRI #2, Iowa State University, Ames, IA 50011, USA

## Abstract

**Background:**

Suppression subtractive hybridization (SSH) strategy was used with extraintestinal pathogenic *Escherichia coli *(EXPEC) that cause avian colibacillosis (avian pathogenic *E. coli *or APEC) and human urinary tract infections (uropathogenic *E. coli *or UPEC) to determine if they possessed genes that were host and/or niche specific. Both APEC and UPEC isolates were used as tester and driver strains in 4 different SSHs in order to obtain APEC- and UPEC-specific subtraction fragments (SFs).

**Results:**

These procedures yielded a total of 136 tester-specific SFs of which 85 were APEC-derived and 51 were UPEC-derived. Most of the APEC-derived SFs were associated with plasmids; whereas, the majority of UPEC-derived sequences matched to the bacterial chromosome. We further determined the distribution of these tester-derived sequences in a collection of UPEC and APEC isolates using polymerase chain reaction techniques. Plasmid-borne, APEC-derived sequences (*tsh*, *cva*B, *tra*R, *tra*C and *sop*B) were predominantly present in APEC, as compared to UPEC. Of the UPEC-derived SFs, those encoding hemolysin D and F1C major and minor fimbrial subunits were present only in UPEC. However, two UPEC-derived SFs that showed strong similarity to the uropathgenic-specific protein gene (*usp*) occurred in APEC, demonstrating that *usp *is not specific to UPEC.

**Conclusion:**

This study provides evidence of the genetic variability of ExPEC as well as genomic similarities between UPEC and APEC; it did not identify any single marker that would dictate host and/or niche specificity in APEC or UPEC. However, further studies on the genes that encode putative or hypothetical proteins might offer important insight into the pathogenesis of disease, as caused by these two ExPEC.

## Background

Extraintestinal pathogenic *Escherichia coli *(ExPEC) are a specific group of *E. coli *that cause a diverse spectrum of invasive infections in animals and humans often leading to septicemia [1,2]. Among the typical extraintestinal infections caused by ExPEC in humans are urinary tract infections (UTIs), which are a major public health concern in developed countries costing healthcare systems billions of dollars annually [3-5]. Similarly, colibacillosis, caused by avian ExPEC isolates (avian pathogenic *E. coli *or APEC), is an economically devastating disease to poultry industries worldwide [1,6].

Both APEC and human ExPEC, implicated in UTIs (uropathogenic *E. coli *or UPEC), are similar in that they both possess a common set of virulence markers such as various adhesins, iron uptake systems, complement resistance traits, and invasins [2,7-17]. However, no single virulence factor has been shown to be specifically unique to, or definitive of, UPEC or APEC, suggesting that these ExPEC might lack host specificity. Intriguingly, some of the virulence genes that occur on APEC's plasmids (aerobactin, salmochelin, and *sit *operons) and pathogenicity islands (PAIs) (*pap *operon that encodes P fimbriae and *kps *gene cluster) also occur on plasmids and PAIs of UPEC [8,10,13-16,18]. A growing body of evidence suggests that APEC could be a possible source of UPEC causing UTIs or other diseases in human beings [10,16,19-24]. Similarly, *E. coli *plasmids may serve as reservoirs of resistance or virulence genes for human ExPEC [19,20,23], and APEC plasmids contribute to uropathogenicity of *E. coli *in mice [25]. On the other hand, UPEC and APEC may be armed with specific genes that determine their specificity to a particular host (human vs. avian) or niche (e.g., urinary tract vs. respiratory tract).

To better evaluate the relationship between APEC and UPEC, a comparative analysis of APEC and UPEC genomes is important. Such studies might also clarify evolutionary relationships between these two ExPECs and identify genes that decide vital differences in virulence and host specificity. Several PCR-based DNA subtraction methods have been used for the detection of genetic differences between two closely related genomes and subsequent identification of genes responsible for bacterial virulence [26,27]. Here, we describe the use of genomic suppressive subtractive hybridization (SSH) to compare APEC and UPEC strains in order to determine if they harbor host and/or site-specific DNA sequences. Four SSHs were run in the present study using two APEC isolates and two UPEC isolates. Well characterized APEC O1 and UPEC CFT073 strains were included in the study to determine the known APEC- and UPEC-specific sequences that may involved in the disease process [[13,14,17,21,26,28,29], GenBank Accession No. for APEC O1: NC_008563, and for UPEC CFT073: AE014075]. Two other strains, APEC 113 and UPEC 88 that harbor a common set of virulence genes, were included to look for hitherto unknown genes that are implicated in APEC and UPEC pathogenicity [16]. Each APEC and UPEC strain was used as both driver and tester strains in order to obtain both APEC- and UPEC-specific subtraction fragments (SFs). Additionally, we examined a collection of APEC and UPEC isolates with PCR to understand the distribution of these SFs among ExPEC.

## Results and discussion

### Characteristics of the strains used for the study

Several methods such as the embryo lethality assay (ELA) and the day-old chicken challenge model using intratracheal (IT), subcutaneous (SC), intravenous (IV) and intramuscular (IM) routes of inoculation have been used extensively to assess the virulence of avian *E. coli *[30-32]. The ELA is a simple method that can be used to discriminate between virulent and avirulent APEC strains [31]. We used this method to characterize the four isolates that served as driver and tester strains in this study. As determined by chick embryos challenge, APEC O1, APEC 113, UPEC CFT073 and UPEC 88 were identified as virulent (Table 1). While ELA results correlate with that of SC, IV and IM day-old challenge models, they do not correlate with the IT challenge model [30,33]. Therefore, we further characterized these APEC strains with the IT challenge model [32]. This model, which categorized APEC into 3 groups, highly pathogenic, intermediate pathogenic or low pathogenic on the basis of deaths and macroscopic lesions, demonstrated that APEC O1 is highly pathogenic while APEC 113 falls in the intermediate pathogenic group (Table 1).

**Table 1:** Characteristics of tester and driver strains

	**MLST^A^**				
				
**Strain**	**ST^B^**	**ST complex**	**Phylogenetic group^C^**	**% Embryo deaths^D^**	**Pathogenicity group^E^**
APEC O1	95	95	B2	60	High
APEC 113	79	95	B2	15	Intermediate
UPEC 88	73	73	B2	45	NA
UPEC CFT073	73	73	B2	40	NA

^A^MLST, multilocus sequence typing.

^B ^ST, sequence type.

^C ^*E. coli *isolates in phylogenetic groups B2 and D are more likely to be associated with disease than phylogenetic groups A and B1.

^D^20 eggs were inoculated for each isolate. There were no deaths in the control groups (PBS-inoculated and uninoculated eggs).

^E^Pathogenicity group of the 2 APEC strains were determined according to a previously described scheme [32]. Highly pathogenic (high) isolates caused mortality or severe lesions (pericarditis, perihepatitis, and/or liver necrosis) in >50% of the chickens inoculated. Intermediate pathogens did not cause death and produced lesions in <50% of chickens inoculated. Weak pathogens (low) produced no mortality and occasional airsacculitis NA – not applicable, because pathogenicity of UPEC isolates for day-old chickens was not determined.

Phylogenetic analysis using triplex PCR has shown that *E. coli*strains can be grouped into four main phylogenetic groups, namely, A, B1, B2, and D. Virulent ExPEC strains are said to belong mainly to group B2 and, to a lesser extent, to group D, whereas most commensal *E. coli *strains belong to group A [34]. As shown in Table 1, all four *E. coli *strains used for SSH belonged to the B2 phylogenetic group. Of the 95 UPEC isolates used for the gene prevalence studies, the majority (79%) fell into one of the virulence-associated phylogenetic groups, B2 (61%) or D (18%); whereas, only 16% belonged to the other two phylogenetic groups (Table 2). However, of the 95 APEC isolates used for gene prevalence studies, 36%, 11%, 22% and 26% of strains belonged to phylogenetic groups A, B1, B2 and D, respectively (Table 2). Several recent studies reported similar results for APEC, suggesting that predictions about the virulence of APEC strains cannot be based merely on chromosomal differences, as used in this typing procedure [10,16]. Plasmid PAIs have a strong association with APEC's capacity to cause disease and will likely need to be given due consideration when typing APEC isolates [13,16,17,25,28].

**Table 2:** Phylogenetic groups of the *E. coli *isolates used in the SF distribution study

	**Number of isolates in each phylogenetic group (%)^B^**			
	
**Category of *E. coli*^A^**	**A**	**B1**	**B2**	**D**
**APEC**	36 (37.89)	11 (11.58)	22 (23.16)	26 (27.37)
**UPEC**	10 (10.53)	6 (6.32)	61 (64.21)	18 (18.94)

^A^Each category of *E. coli *contains 95 isolates. APEC, Avian Pathogenic *Escherichia coli*; UPEC, Uropathogenic *Escherichia coli*.

^B^Percentages of isolates falling within each phylogenetic group are shown in parentheses. *E. coli *isolates in phylogenetic groups B2 and D are more likely to be associated with disease than phylogenetic groups A and B1 [34].

**Figure 1:**
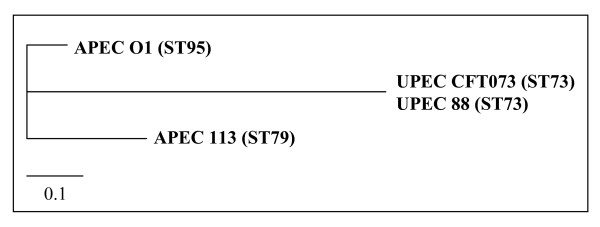
Unrooted phylogram (neighbor-joining tree) of MLST loci of APEC and UPEC strains used for SSHs. The tree was constructed from the concatenated sequences of the 7 MLST loci described in the text. Branch lengths reflect absolute nucleotide differences between concatenated sequences.

Multilocus sequence typing (MLST) provides a novel approach to molecular epidemiology and strengthens our understanding of phylogenetic distribution of infectious disease agents [35]. Further, MLST data can be transferred between laboratories around the globe via the web-accessible databases. MLST of driver and tester strains revealed that the two APEC strains belong to the ST95 complex and two UPEC strains belong to the ST73 complex as defined by the publicly available *E. coli *MLST database (Figure 1, Table 1). By comparison with this database, the two UPEC and two APEC strains used as driver and tester strains in this study were found to be phylogenetically related to each other.

### Sequence analysis of tester-specific fragments

Four subtracted libraries of tester strains were constructed for four different SSHs with the aim of identifying genomic differences between APEC and UPEC. Four SSHs yielded a total of 482 tester-specific clones. After amputation of the vector sequences and regions of low quality (Phred quality value < Q20), 258 sequences (≥ 100 bp in size) remained and were regarded as valid SFs. Of these 258 SFs, 122 sequences were discarded due to redundancy (overlapping ≥ 90% and similarity ≥ 98%) or because they were present in the *E. coli *K12 genome. The remaining 136 SFs ranged in size from 121–1343 bp. Using the BLAST program, a search for similarity with these sequences was undertaken (see Additional files 1, 2, 3, 4). Additionally, these SFs were used as targets in subsequent sequence prevalence studies. Of the 136 tester-specific SFs, 46 were APEC O1-derived (SSH1), 28 were UPEC CFT073-derived (SSH2), 39 were APEC 113-derived (SSH3), and 23 were UPEC 88-derived (SSH4). The SFs were further categorized as sequences that have previously described functions; similarity to phage/prophage sequences; encode hypothetical proteins; or lack similarity to any of the genes in published databases. SSH1 yielded 10 SFs that corresponded to genes with known functions (4 plasmid-associated SFs, and 6 chromosomal-associated SFs), 22 SFs that are parts of genes with putative or unknown functions (8 plasmid-associated SFs, and 14 chromosomal-associated SFs including 5 phage-derived sequences), and 14 SFs that are unique to APEC O1 strain. Of the 28 CFT073-derived SFs obtained from SSH2, 22 sequences corresponded to genes that encode hypothetical proteins and another 6 to the genes encoding proteins with known functions. All 28 CFT073-borne SFs were located on the chromosome including 2 that showed similarity to phages or prophages. Among the SFs of SSH3, 6 fragments did not show similarity to any known sequences, 9 showed similarity to genes with known functions (8 plasmid-associated SFs, and 1 chromosomal-associated), and 24 showed similarity to genes with unknown or putative functions (3 plasmid-associated SFs, and 21 chromosomal-associated SFs including 2 phage-borne sequences). Of the 23 SFs obtained from SSH4, 15 chromosomal-located SFs showed similarity to genes with hypothetical functions, 6 had similarity to genes with known functions (4 plasmid-located SFs, and 2 chromosomal-located SFs), while 1 sequence was unique to the driver strain UPEC 88. Interestingly, 23 APEC-derived SFs matched to plasmid regions, where only 5 of UPEC-derived fragments did, suggesting that plasmids may play a more prominent role in APEC virulence than they do in UPEC. Further, the presence of plasmid sequences, phage/prophage sequences, integrases, recombinases, and transposases among the SFs strongly support the already established theory of evolution of bacterial pathogenicity through horizontal gene transfer and genetic recombination

### Prevalence of SFs among APEC and UPEC

The prevalence of SFs among a collection of APEC and UPEC is summarized (see Additional files 1, 2, 3, 4). There was no statistically significant difference between the prevalence of 66 APEC-derived SFs among APEC and UPEC out of a total of 85 APEC-derived SFs. None of the APEC-derived fragments that were present in more than 10% of APEC tested was limited only to APEC. However, certain APEC O1-derived SFs could be traced back to a PAI located on a large plasmid in APEC O1, pAPEC-O1-ColBM [28]. These fragments included *tra*A and *tra*C (SF A12), *tsh *(SF A22), and *sopB *(SF A28) occurred significantly more often in APEC than in UPEC. Similarly, the APEC 113-derived SF B11 that matched *cvaB *of pAPEC-O2-ColV was present in 63% of the APEC, while only 7% of UPEC carried the sequence [13]. The *cvaB *is a gene located in the ColV operon [20], and *tra *genes encode for plasmid transfer proteins [13,28]. The *tsh*, which encodes the temperature-sensitive hemagglutinin (Tsh), is involved in development of air sac lesions of birds during colibacillosis [36], highly prevalent among APEC, carried by highly pathogenic strains of APEC, and considered to be an APEC virulence marker [17,36,37]. However, a previous report indicates that a gene homologue to *tsh *is present in UPEC CFT073 though it is less conserved relative to its counterpart in APEC [38]. The APEC-O1 derived SF A22 obtained from SSH1 showed 100% homology to a region of *tsh *of APEC-O1-ColBM but matched to a region that is less conserved in *tsh *homologies from other pathogens. Interestingly, APEC 113-derived fragments, B27 and B28, which showed 100% similarity to two different regions of the putative phosphotransferase system encoded by an APEC GimB genetic island, were present predominantly in UPEC (37%) rather than in APEC (10%) (GenBank AJ810519).

In contrast, most of the UPEC-derived fragments were present at a higher rate in UPEC than in APEC. Yet, many of these SFs contained genes that encode putative or hypothetical proteins, making it difficult to directly relate them to UPEC pathogenicity. Functional assays coupled with construction of isogenic mutants of such genes followed by subsequent testing in experimental infection models will likely provide new insight into UPEC pathogenicity and lead to discovery of previously unknown UPEC virulence mechanisms. Intriguingly, the SFs, U14, U20/U27, which matched to the *foc *gene cluster, encoding the F1C fimbrial major and minor subunit precursors, and U28, which corresponds to the *hlyD *gene, were exclusively present in UPEC. F1C fimbriae, which lack hemagglutination properties, are known to mediate specific adherence of UPEC to the collecting ducts and distal tubules of the human kidney [39]. The *hlyD *gene, a well known UPEC virulence marker, is present on PAIs in at least in some UPEC strains [8,40]. The *hly *operon of UPEC consists of four genes: *hlyA*, *hlyB*, *hlyC*, and *hlyD *[41]. Although this operon is known to be present in CFT073 and thought to be absent in these APEC strains, this study did not detect any UPEC-derived sequences with similarity to *hly *genes other than *hlyD*, perhaps due to inappropriate fragment sizes yielded with the restriction enzymes used in the present study. Two UPEC 88-derived fragments, C19 and C22, which matched to regions of the *usp *gene that encodes the uropathogenic-specific protein, were present predominantly in UPEC rather than in APEC. Although *usp *was thought to be uropathogenic-specific and a virulence marker of UPEC [42], a small percentage of APEC contained the gene revealing that it is not strictly specific to UPEC.

Previously, we carried out SSH between APEC O1 and a commensal *E. coli *strain isolated from feces of a healthy chicken [26]. This study demonstrated that genes, encoding the Tia invasion determinant protein, the iron-responsive element (IreA), P pili, and aldo-keto reductase are more common in APEC and UPEC than in avian commensal *E. coli*. However, neither this study, nor the present one, detected a single trait that was unequivocally present in all the UPEC or all the APEC tested. Had such traits been detected, we would have suspected that they were involved in host and/or niche specificity of these two ExPEC. We suggest that delineating the functions of hypothetical and unknown proteins of UPEC and APEC would strengthen this conclusion and add to our current understanding of ExPEC pathogenesis.

To the authors' knowledge, this is the first study comparing APEC and UPEC genomes using SSH to explore their differences and similarities and to identify APEC- and UPEC-specific genes that may be involved in ExPEC pathogenicity in different hosts and niches. When this study was conducted, the genome sequence of APEC O1 (an O1:K1:H7 strain) was not completed. Since the genome sequence of this strain is now publicly available, direct comparison of APEC O1 and human ExPEC genomes (e.g., UPEC CFT073) can be used to facilitate identification of APEC- and UPEC-specific genes. However, it is remarkable that the present study identified 14 APEC O1-specific, hitherto unknown sequences (30% of APEC-derived SFs) that are absent from the other APEC isolates tested.

## Conclusion

SSH between APEC and UPEC identified some genes that are already known to be associated with the virulence of these two pathogens. Some of the UPEC-derived genes encode putative or hypothetical proteins. Delineation of their functions might reveal factors that determine host and/or niche specificity. This study also confirmed the findings of others that APEC virulence is commonly associated with plasmid-linked genes [13,16,17,25,28]. None of the SFs were present in almost all the isolates screened, and it is apparent that both pathogens use a combination of virulence factors to establish disease in the host.

## Methods

### Bacterial strains and growth conditions

Two UPEC strains, CFT073 and UPEC 88, were used for SSHs. These were kindly supplied by Dr. James Johnson (Mucosal Vaccine Research Center, VA Medical Center, and Department of Medicine, University of Minnesota, Minneapolis, MN) and Dr. Paul Carson (Meritcare Hospital, Fargo, ND), respectively. The CFT073 strain (O6 serogroup) originated from the blood of a woman with pyelonephritis [29,43], and UPEC 88 (O6 serogroup) was isolated from the urine of a patient with cystitis [16]. Two APEC strains, APEC O1 (O1 serogroup) and APEC 113 (O2 serogroup), used for SSHs, were isolated from the lung and bone marrow of two different turkeys with colisepticemia [16,26]. An additional collection of 95 APEC and 95 UPEC isolates were used to study the distribution of SFs in ExPEC using the polymerase chain reaction (PCR). These additional APEC and UPEC isolates have been described previously [16,26]. Additional APEC isolates originated from chickens and turkeys having lesions of colibacillosis; whereas, the additional UPEC isolates originated from cases of human UTIs and were kindly provided by Dr. Paul Carson (Meritcare Hospital, Fargo, ND). Strains were grown routinely at 37°C overnight in Luria-Bertani (LB) broth and LB agar. When necessary, media were supplemented with 100 μg ampicillin ml^-1^. All strains were stored frozen at -80°C in Brain Heart Infusion (BHI) broth with 20% (v/v) glycerol.

### Characterization of driver and tester strains

Virulence of the *E. coli *isolates used in SSHs was determined in embryonated eggs as described previously [31]. In brief, each isolate was grown in BHI broth overnight at 37°C, washed twice in phosphate-buffered saline (PBS), resuspended in PBS and diluted to approximately 10^6 ^cells ml^-1 ^PBS. After quantifying the bacterial concentration by viable counts, 0.1 ml of the diluted culture was inoculated into the allantoic cavity of 12-day-old, specific-pathogen-free (SPF) eggs. Eggs were candled once daily for 4 days post-infection, and the deaths were recorded. PBS-inoculated and uninoculated SPF eggs were included as controls.

The two APEC isolates used for SSHs were assigned to pathogenicity groups according to the method described previously [32]. Briefly, two groups of broiler chickens (6 chickens in a group) were inoculated with 0.1 ml of the appropriate bacterial suspension in PBS containing 10^7 ^cells ml^-1 ^by the intratracheal route. The pathogenicity group of each strain was determined by comparison of the mortalities and macroscopic lesions they caused to that seen in birds inoculated with APEC isolates of known pathogenicity groups. Isolates for comparison belonging to high, intermediate and low pathogenicity groups were kindly supplied by Dr. Sandra Cloud (University of Delaware, Newark, DE). Another group received 0.1 ml of PBS and served as a placebo control.

Bacterial strains used for SSHs were subjected to MLST to understand the phylogenetic relationship between driver and tester strains as previously described [35]. Briefly, 583 to 932 bp internal fragments of seven housekeeping gene loci in the *E. coli *chromosome (*adk *– adenylate kinase; *fumC *– fumarate hydratase; *gyrB *– DNA gyrase; *icd *– isocitrate/isopropylmalate dehydrogenase; *mdh *– malate dehydrogenase; *purA *– adenylosuccinate dehydrogenase; *recA *– ATP/GTP-binding motif) were amplified by PCR, and the sequence type (ST) and ST complex of the strain were defined according to the *E. coli *MLST data base maintained at the Max-Planck Institut fuer Infektionsbiologie [44].

All the *E. coli *used in this study were subjected to phylogenetic analysis according to the previously published scheme [34]. Briefly, a triplex PCR was employed to amplify the two genes, *chuA *and *yiaA*, and the DNA fragment, TSPE4. Based on these results, the isolates were assigned to one of four groups (A, B1, B2 and D).

### Genomic suppression subtractive hybridization

Four SSHs, namely, SSH1, SSH2, SSH3 and SSH4 were carried out using two strains of APEC and two strains of UPEC. SSH1 and SSH2 were carried out between APEC O1 strain and UPEC CFT073 strain using APEC O1 and UPEC CFT073 as the tester strain, respectively. In order to enhance the probability of identifying hitherto unknown genes involved in APEC and UPEC pathogenicity, SSH3 and SSH4 were carried out between APEC 113 and UPEC 88 which share an identical genetic profile based on the known virulence genes [16]. The Clontech PCR-Select Bacterial Genome Subtraction Kit (ClontechLaboratories, Inc., Palo Alto, CA) was used for the SSHs according to the manufacturer's instructions. Briefly, the tester and driver genomic DNA used for each SSH was digested with the same four-base cutting restriction enzymes (*Rsa*I or *Hae*III). The tester DNA was then aliquoted into two tubes, and the DNA in each aliquot was ligated to a different adaptor provided with the kit (adaptors 1 and 2R). Two hybridizations were carried out in the presence of excess driver DNA. The product of the second hybridization was then used as template in a PCR reaction for enrichment of the tester-specific sequences. The amplified PCR products were subsequently cloned into the pGEM T-Easy vector (Promega, Madison, WI) and transformed into competent *E. coli *JM109 (Promega). The subtracted library was screened for tester-specific SFs exactly according to the method described previously by Kariyawasam *et al*. [26], using the DIG High Prime Labeling and Detection Starter Kit™ (Roche Diagnostics, Penzberg, Germany). Tester-derived libraries were analysed with the PHRED program (University of Washington, Seattle, WA) to identify the miscalled bases [45]. Sequences having a PHRED quality score of at least 20 were considered of good quality and were taken for further analysis.

### DNA sequencing and bioinformatics

Tester-specific clones were grown in LB containing ampicillin, and the recombinant plasmids were purified using Plasmid Minipreps from Promega. Inserts were sequenced bi-directionally at the DNA Sequencing and Synthesis Facility at Iowa State University, Ames, IA, using the BigDye terminator chemistry (Applied Biosystems, Foster City, CA). The BLASTN and BLASTX searches were performed on the National Center for Biotechnology Information website to identify the genes from the subtraction library [46].

#### Nucleotide sequence accession numbers

The nucleotide sequences of the UPEC or APEC-specific fragments (see Additional files 1, 2, 3, 4) have been submitted to GenBank under the accession numbers DQ988883–DQ988928, ED797564–ED797572, ED797582–ED797590, ED797599–ED797606, ED797616–ED797625, ED797573–ED797581, ED797591–ED797598, ED797608–ED797615, and EI415524–EI415497.

#### Prevalence of SFs in APEC and UPEC

Oligonucleotide primer sets were designed (Primer 3 software) to amplify the tester-specific sequences obtained from the subtractive hybridization library and procured from Integrated DNA Technologies, Commercial Park, Coralville, IA. An overview of the primers used andthe expected amplicon sizes are shown in Additional file 5. Each 25-μl PCR reaction mixture contained 2.5 μl of 10 × PCR buffer (100 mM Tris-HCl, pH 8.4, and 500 mM KCl), 0.25 μl of 250 mM MgCl_2_, 0.40 μl of 10 mM deoxynucleoside triphosphates, 0.5 μl of each of the forward and reverse primers (stock concentration, 20 μM), 0.1 μl (5 U μl^-1^) of *Taq *DNA polymerase (Invitrogen), 2 μl of template DNA extracted by the rapid boiling method, and 18.75 μl of sterile double distilled water. After denaturation at 94°C for 3 min, the sampleswere subjected to 30 cycles of 94°C for 45 s, 59°C for 45 s, and 72°C for 45 s, followed by final 5-min incubation at 72°C. Samples were fractionated by 1.5% (w/v) agarose gel electrophoresis and visualized by ethidium bromide staining.

#### Biostatistics

Prevalence data for each of the tester-specific SHFs were analyzed by two-tailed Fisher's exact test, controlling the multiple comparison error rates by the Bonferroni method [47]. Analyses were conducted with a standard statistical software (GraphPad Software, Inc, San Diego, CA).

## Authors' contributions

SK conceived the study, carried out molecular genetic studies, sequence alignment and analysis of data, and drafted the manuscript. JS participated in running the polymerase chain reactions. LKN participated in the design and analysis of data, coordinated the study, and revised the manuscript critically. All the authors have read and approved the final manuscript.

## Supplementary Material

Additional file 1 Summary of BLAST search results for SFs obtained as a result of SSH between APEC O1 (tester strain) and UPEC CFT073 (driver strain). The data provided represent the BLAST search results for SFs obtained with SSH between APEC O1 (tester strain) and UPEC CFT073 (driver strain), and statistical comparison of occurrence of those SFs among a collection of APEC and UPEC. Click here for file

Additional file 2 Summary of BLAST search results for SFs obtained as a result of SSH between UPEC CFT073 (tester strain) and APEC O1 (driver strain). The data provided represent the BLAST search results for SFs obtained with SSH between UPEC CFT073 (tester strain) and APEC O1 (driver strain), and statistical comparison of occurrence of those SFs among a collection APEC and UPEC. Click here for file

Additional file 3 Summary of BLAST search results for SFs obtained as a result of SSH between APEC 113 (tester strain) and UPEC 88 (driver strain). The data provided represent the BLAST search results for SFs obtained with SSH between APEC 113 (tester strain) and UPEC 88 (driver strain), and statistical comparison of occurrence of those SFs among a collection of APEC and UPEC. Click here for file

Additional file 4 Summary of BLAST search results for SFs obtained as a result of SSH between UPEC 88 (tester strain) and APEC 113 (driver strain). The data provided represent the BLAST search results for SFs obtained with SSH between UPEC 88 (tester strain) and APEC 113 (driver strain), and statistical comparison of occurrence of those SFs among a collection of APEC and UPEC. Click here for file

Additional file 5 Primers used in the SF prevalence study. This table shows the primer sequences that were used to PCR amplify tester-specific sequences obtained from four different SSHs. Click here for file
