# Supplementary material for: Common and specific genomic sequences of avian and human extraintestinal pathogenic Escherichia coli as determined by genomic subtractive hybridization
Source: BMC Microbiol. 2007 Aug 30;7:81. doi: 10.1186/1471-2180-7-81 (PMC2031896; doi:10.1186/1471-2180-7-81)
Supplement: Additional file 1 — Summary of BLAST search results for SFs obtained as a result of SSH between APEC O1 (tester strain) and UPEC CFT073 (driver strain). The data provided represent the BLAST search results for SFs obtained with SSH between APEC O1 (tester strain) and UPEC CFT073 (driver strain), and statistical comparison of occurrence of those SFs among a collection of APEC and UPEC. [file 1471-2180-7-81-S1.doc]

**Summary of BLAST search results for SFs obtained as a result of SSH between APEC O1 (tester strain) and**

**UPEC CFT073 (driver strain)**

| **Location of SF** | **SF** | **Size**  **(bp)** | **Translated products of the nucleotide sequences with similarity^A^** | **GenBank accession** | **% identity** | **% of positive isolates^B^** | |
| --- | --- | --- | --- | --- | --- | --- | --- |
|  |  |  |  |  |  | **APEC** | **UPEC** |
| **Plasmid** | A1 | 433 | A conserved hypothetical protein of plasmid pAPEC-O1-R of APEC strain O1:K1 | [DQ517526](http://www.ncbi.nlm.nih.gov/entrez/viewer.fcgi?db=nucleotide&val=99867038) | 100 | 6 | 0 |
|  | A2 | 692 | Putative inner membrane protein of plasmid pAPEC-O1-R of APEC strain O1:K1 | [DQ517526](http://www.ncbi.nlm.nih.gov/entrez/viewer.fcgi?db=nucleotide&val=99867038) | 100 | 7 | 9 |
|  | A3 | 604 | ISEc12 putative ATP-binding protein of plasmid pAPEC-O1-ColBM of APEC | DQ381420 | 100 | 30^*^ | 10^*^ |
|  | A9 | 245 | IS1 InsA protein, and *E. coli* transport system, efflux protein EtsC of plasmid pAPEC-O1-ColBM of APEC strain O1:K1 | DQ381420 | 100 | 10 | 1 |
|  | A12 | 530 | Conjugal transfer protein, (TraR), and a protein for pilus assembly and synthesis (TraC) of plasmid pAPEC-O1-ColBM of APEC strain O1:K1 | DQ381420 | 99 | 72^*^ | 15^*^ |
|  | A13 | 453 | Putative ribonucleoside-diphosphate reductase alpha subunit of *Yersinia pestis* biovar *Microtus* strain 91001 plasmid pMT1 | AE017045 | 78 | 9 | 1 |
|  | A20 | 511 | Putative exported protein of plasmid pAPEC-O1-R of APEC O1:K1 | DQ517526 | 99 | 0 | 0 |
|  | A22 | 254 | Temperature-sensitive hemagglutinin (Tsh) of APEC plasmid pAPEC-O1-ColBM | DQ381420 | 100 | 54^*^ | 2^*^ |
|  | A27 | 265 | Puttive transposase of plasmid pAPEC-O1-ColBM of APEC strain O1:K1 | DQ381420 | 98 | 16 | 11 |
|  | A28 | 580 | SopB (stabilization of plasmid protein) of plasmid pAPEC-O1-ColBM of APEC O1:K1 | DQ381420 | 99 | 43^*^ | 8^*^ |
|  | A35 | 559 | A region between putative pilus assembly protein (TrhC) and a putative plasmid partitioning protein (ParA)of pAPEC-O1-R of APEC O1:K1 | DQ517526 | 97 | 7 | 0 |
|  | A48 | 389 | A conserved hypothetical protein of APEC plasmid  pAPEC-O1-ColBM | [DQ381420](http://www.ncbi.nlm.nih.gov/entrez/viewer.fcgi?db=nucleotide&val=88770133) | 100 | 25 | 22 |
| **Chromosome** | A6 | 527 | A conserved hypothetical protein of *Shigella flexneri* 2a strain 301 | AE005674 | 94 | 12 | 19 |
|  | A7 | 301 | Endo-alpha-sialidase of UPEC strain UTI89 | CP000243 | 99 | 10 | 17 |
|  | A8 | 604 | Putatitive transposase subunit of PAI I_APEC-O1_ of APEC strain O1:K1 | DQ095216 | 100 | 30^*^ | 10^*^ |
|  | A10 | 387 | Endo-alpha-sialidase and an antirepressor protein of UPEC strain UTI89 | CP000243 | 93 | 13 | 14 |
|  | A11 | 508 | Putative glycoxylate carboligase (GclA) of UPEC strain UTI89 | CP000243 | 99 | 20 | 21 |
|  | A16 | 401 | A hypothetical protein of a prophage of UPEC strain UTI89 | CP000243 | 99 | 2 | 11 |
|  | A17 | 377 | A conserved hypothetical protein of APEC O1:K1 strain | NC_008563 | 100 | 27 | 12 |
|  | A19 | 262 | Putative transposase subunit of PAI I_APEC-O1_ of APEC strain O1:K1 | DQ095216 | 99 | 15 | 11 |
|  | A23 | 292 | [Antitermination protein gp23](http://www.ncbi.nlm.nih.gov/entrez/viewer.fcgi?val=91070629&db=Nucleotide&from=2623228&to=2623746&view=gbwithparts) and a [hypothetical protein](http://www.ncbi.nlm.nih.gov/entrez/viewer.fcgi?val=91070629&db=Nucleotide&from=2623743&to=2623931&view=gbwithparts) of bacteriophage ST64T UPEC strain UTI89 | CP000243 | 100 | 13 | 13 |
|  | A29 | 296 | Tia invasion determinant protein of PAI I_APEC-O1_ of APEC strain O1:K1 | DQ095216 | 100 | 32 | 29 |
|  | A30 | 705 | Putative transposase subunit of PAI I_APEC-O1_ of APEC strain O1:K1 | DQ095216 | 99 | 15 | 11 |
|  | A31 | 526 | Hypothetical protein of UPEC strain UTI89 | CP000243 | 95 | 16 | 18 |
|  | A32 | 337 | Superfamily I DNA helicase of PAI I_APEC-O1_ of APEC strain O1:K1 | DQ095216 | 100 | 35 | 36 |
|  | A36 | 496 | A conserved hypothetical protein and RecT-like protein of APEC strain O1:K1 | NC_008563 | 100 | 4 | 8 |
|  | A39 | 605 | E[nterobacteria phage Sf6 gene 63 protein](http://www.ncbi.nlm.nih.gov/entrez/viewer.fcgi?val=91070629&db=Nucleotide&from=2926523&to=2926960&view=gbwithparts) and a [putative endolysin of prophage CP-933X](http://www.ncbi.nlm.nih.gov/entrez/viewer.fcgi?val=91070629&db=Nucleotide&from=2926957&to=2927433&view=gbwithparts) of UPEC strain UTI89 | CP000243 | 99 | 15 | 17 |
|  | A41 | 467 | Putative bacteriophage protein of of *Shigella flexneri* 5b strain 8401 | CP000266 | 96 | 1 | 6 |
|  | A42 | 361 | A conserved hypothetical protein of APEC O1:K1 strain | NC_008563 | 100 | 27 | 12 |
|  | A43 | 216 | [Putative tail fiber protein](http://www.ncbi.nlm.nih.gov/entrez/viewer.fcgi?val=47118301&db=Nucleotide&from=1649515&to=1652430&view=gbwithparts) of *E. coli* O157:H7 str. Sakai DNA | BA000007 | 78 | 2 | 0 |
|  | A44 | 539 | Endo-alpha-sialidase of UPEC strain UTI89 | CP000243 | 99 | 10 | 17 |
|  | A47 | 591 | P[olypeptide destructive to membrane potential](http://www.ncbi.nlm.nih.gov/entrez/viewer.fcgi?val=73854091&db=Nucleotide&from=2000469&to=2000624&view=gbwithparts) of *S. sonnei* strain Ss046 | CP000038 | 96 | 12 | 8 |
| **Other** | A4 | 547 | unique |  |  | 0 | 0 |
|  | A5 | 682 | unique |  |  | 0 | 0 |
|  | A14 | 666 | A unique region and a 185-bp region corresponds to a hypothetical protein of phage phiV10 | DQ126339 | 92 | 9 | 0 |
|  | A15 | 321 | Unique |  |  | 0 | 1 |
|  | A18 | 299 | Unique |  |  | 0 | 1 |
|  | A21 | 670 | Unique |  |  | 0 | 0 |
|  | A25 | 448 | Unique |  |  | 0 | 0 |
|  | A26 | 298 | Unique |  |  | 0 | 1 |
|  | A33 | 241 | Unique |  |  | 0 | 1 |
|  | A34 | 504 | Unique |  |  | 0 | 0 |
|  | A37 | 624 | Unique |  |  | 0 | 1 |
|  | A38 | 492 | Unique |  |  | 0 | 1 |
|  | A40 | 397 | Unique |  |  | 2 | 1 |
|  | A46 | 444 | Unique |  |  | 0 | 1 |

^A^ Note that the SFs represented only portions of individual genes or genetic elements and were by no means complete gene sequences. Some SFs represent different regions of the same gene and so have identical translated products. The SFs categorized under “other” are absent in the published databases including the APECO1:K1 genome (NC_008563).

^B^ Each category of *E. coli* contains 95 isolates. ^*^ indicates a statistically significant difference (*P*< 0.001, Fisher’s exact test, with Bonferroni adjustment).
