# Supplementary material for: Common and specific genomic sequences of avian and human extraintestinal pathogenic Escherichia coli as determined by genomic subtractive hybridization
Source: BMC Microbiol. 2007 Aug 30;7:81. doi: 10.1186/1471-2180-7-81 (PMC2031896; doi:10.1186/1471-2180-7-81)
Supplement: Additional file 2 — Summary of BLAST search results for SFs obtained as a result of SSH between UPEC CFT073 (tester strain) and APEC O1 (driver strain). The data provided represent the BLAST search results for SFs obtained with SSH between UPEC CFT073 (tester strain) and APEC O1 (driver strain), and statistical comparison of occurrence of those SFs among a collection APEC and UPEC. [file 1471-2180-7-81-S2.doc]

**Summary of BLAST search results for SFs obtained as a result of SSH between UPEC CFT073 (tester strain) and**

**APEC O1 (driver strain)**

| **Location of SF** | **SF** | **Size**  **(bp)** | **Translated products of the nucleotide sequences with similarity^A^** | **GenBank accession** | **% identity** | **% of positive isolates^B^** | |
| --- | --- | --- | --- | --- | --- | --- | --- |
|  |  |  |  |  |  | **APEC** | **UPEC** |
| **Chromosome** | U1 | 384 | A hypothetical protein of UPEC strain CFT073 | AE014075 | 100 | 18^*^ | 53^*^ |
|  | U2 | 702 | R6-like protein of PAI II of UPEC strain CFT073 | AF447814 | 99 | 0^*^ | 42^*^ |
|  | U3 | 618 | A hypothetical protein of UPEC strain CFT073 | AE014075 | 100 | 9 | 18 |
|  | U4 | 691 | A hypothetical protein of UPEC strain CFT073 | AE014075 | 100 | 99^*^ | 37^*^ |
|  | U5 | 663 | [Putative RTX family exoprotein A gene](http://www.ncbi.nlm.nih.gov/entrez/viewer.fcgi?val=26111730&db=Nucleotide&from=340972&to=345804&view=gbwithparts) of UPEC strain CFT073 | AE014075 | 100 | 11 | 22 |
|  | U6 | 307 | A hypothetical protein of UPEC strain CFT073 | AE014075 | 99 | 1 | 8 |
|  | U7 | 678 | A hypothetical protein and p[utative sialic acid transporter](http://www.ncbi.nlm.nih.gov/entrez/viewer.fcgi?val=26111730&db=Nucleotide&from=3476175&to=3477683&view=gbwithparts) of UPEC strain CFT073 | AE014075 | 100 | 0^*^ | 30^*^ |
|  | U8 | 701 | A hypothetical protein of UPEC strain CFT073 | AE014075 | 99 | 2 | 13 |
|  | U9 | 709 | A putative transposase of PAI II of UPEC strain CFT073 | AF447814 | 100 | 4^*^ | 47^*^ |
|  | U10 | 176 | A h[ypothetical protein YhcI](http://www.ncbi.nlm.nih.gov/entrez/viewer.fcgi?val=26111730&db=Nucleotide&from=3477696&to=3478571&view=gbwithparts) of UPEC strain CFT073 | AE014075 | 100 | 0^*^ | 33^*^ |
|  | U11 | 690 | [F1C and S fimbrial switch regulatory protein](http://www.ncbi.nlm.nih.gov/entrez/viewer.fcgi?val=26111730&db=Nucleotide&from=1186872&to=1187156&view=gbwithparts) of UPEC strain CFT073 | AE014075 | 100 | 20 | 23 |
|  | U12 | 451 | A DNA segment between a [putative protein of ShlA/HecA/FhaA exoprotein family](http://www.ncbi.nlm.nih.gov/entrez/viewer.fcgi?val=26111730&db=Nucleotide&from=312645&to=322295&view=gbwithparts) and a hypothetical protein of UPEC strain CFT073 | AE014075 | 100 | 9 | 24 |
|  | U13 | 654 | [DNA adenine methylase](http://www.ncbi.nlm.nih.gov/entrez/viewer.fcgi?val=26111730&db=Nucleotide&from=914250&to=915107&view=gbwithparts) of UPEC strain CFT073 | AE014075 | 99 | 1^*^ | 14^*^ |
|  | U14 | 642 | [F1C fimbrial adhesin precursor](http://www.ncbi.nlm.nih.gov/entrez/viewer.fcgi?val=26111730&db=Nucleotide&from=1193817&to=1194827&view=gbwithparts) and a hypothetical protein of UPEC strain CFT073 | AE014075 | 99 | 0^*^ | 30^*^ |
|  | U15 | 479 | [Putative phage baseplate assembly protein](http://www.ncbi.nlm.nih.gov/entrez/viewer.fcgi?val=26111730&db=Nucleotide&from=929556&to=930134&view=gbwithparts) of UPEC strain CFT073 | AE014075 | 100 | 34 | 26 |
|  | U16 | 607 | A hypothetical protein of UPEC strain CFT073 | AE014075 | 100 | 0 | 1 |
|  | U17 | 548 | A hypothetical protein of UPEC strain CFT073 | AE014075 | 100 | 5^*^ | 53^*^ |
|  | U18 | 361 | A hypothetical protein of UPEC strain CFT073 | AE014075 | 99 | 2^*^ | 50^*^ |
|  | U19 | 475 | A hypothetical protein of UPEC strain CFT073 | AE014075 | 99 | 0^*^ | 48^*^ |
|  | U20 | 364 | [F1C major fimbrial subunit precursor](http://www.ncbi.nlm.nih.gov/entrez/viewer.fcgi?val=26111730&db=Nucleotide&from=1188213&to=1188755&view=gbwithparts) of UPEC strain CFT073 | AE014075 | 100 | 0^*^ | 30^*^ |
|  | U21 | 302 | [Unknown protein encoded by a prophage](http://www.ncbi.nlm.nih.gov/entrez/viewer.fcgi?val=26111730&db=Nucleotide&from=1378670&to=1378936&view=gbwithparts) of UPEC strain CFT073 | AE014075 | 100 | 0 | 10 |
|  | U22 | 561 | [Putative capsid scaffolding protein](http://www.ncbi.nlm.nih.gov/entrez/viewer.fcgi?val=26111730&db=Nucleotide&from=923686&to=924513&view=gbwithparts) and a [hypothetical protein](http://www.ncbi.nlm.nih.gov/entrez/viewer.fcgi?val=26111730&db=Nucleotide&from=924410&to=925702&view=gbwithparts) of UPEC strain CFT073 | AE014075 | 100 | 38 | 27 |
|  | U23 | 464 | P[utative peptide synthetase](http://www.ncbi.nlm.nih.gov/entrez/viewer.fcgi?val=26111730&db=Nucleotide&from=2277138&to=2283602&view=gbwithparts) of UPEC strain CFT073 | AE014075 | 100 | 3^*^ | 35^*^ |
|  | U24 | 393 | A region between two hypothetical proteins of UPEC strain CFT073 | AE014075 | 100 | 2^*^ | 43^*^ |
|  | U25 | 158 | A region between two hypothetical proteins of UPEC strain CFT073 | AE014075 | 99 | 12 | 26 |
|  | U26 | 312 | A hypothetical protein of UPEC strain CFT073 | AE014075 | 100 | 18 | 27 |
|  | U27 | 824 | [F1C minor fimbrial subunit protein G precursor](http://www.ncbi.nlm.nih.gov/entrez/viewer.fcgi?val=26111730&db=Nucleotide&from=1193363&to=1193866&view=gbwithparts) and [F1C putative fimbrial adhesin precursor](http://www.ncbi.nlm.nih.gov/entrez/viewer.fcgi?val=26111730&db=Nucleotide&from=1193817&to=1194827&view=gbwithparts) of UPEC strain CFT073 | AE014075 | 100 | 0^*^ | 30^*^ |
|  | U28 | 354 | Hemolysin D of UPEC strain CFT073 | AE014075 | 100 | 0^*^ | 38^*^ |

^A^ Note that the SFs represented only portions of individual genes or genetic elements and were by no means complete gene sequences. Some SFs represent different regions of the same gene, so have identical translated products.

^B^ Each category of *E. coli* contains 95 isolates. ^*^ indicates a statistically significant difference (*P*< 0.001, Fisher’s exact test, with Bonferroni adjustment).
