# Supplementary material for: Common and specific genomic sequences of avian and human extraintestinal pathogenic Escherichia coli as determined by genomic subtractive hybridization
Source: BMC Microbiol. 2007 Aug 30;7:81. doi: 10.1186/1471-2180-7-81 (PMC2031896; doi:10.1186/1471-2180-7-81)
Supplement: Additional file 3 — Summary of BLAST search results for SFs obtained as a result of SSH between APEC 113 (tester strain) and UPEC 88 (driver strain). The data provided represent the BLAST search results for SFs obtained with SSH between APEC 113 (tester strain) and UPEC 88 (driver strain), and statistical comparison of occurrence of those SFs among a collection of APEC and UPEC. [file 1471-2180-7-81-S3.doc]

**Summary of BLAST search results for SFs obtained as a result of SSH between APEC 113 (tester strain) and**

**UPEC 88 (driver strain)**

| **Location of SF** | **SF** | **Size**  **(bp)** | **Translated products of the nucleotide sequences with similarity^A^** | **GenBank accession** | **% identity** | **% of positive isolates^B^** | |
| --- | --- | --- | --- | --- | --- | --- | --- |
|  |  |  |  |  |  | **APEC** | **UPEC** |
| **Plasmid** | B8 | 300 | Transfer region of plasmid R64 | [AB027308](http://www.ncbi.nlm.nih.gov/entrez/viewer.fcgi?db=nucleotide&val=4903079) | 87 | 0 | 0 |
|  | B11 | 214 | CvaB of plasmid pAPEC-O2-ColV of APEC O2 | [AY545598](http://www.ncbi.nlm.nih.gov/entrez/viewer.fcgi?db=nucleotide&val=83743321) | 100 | 63^*^ | 7^*^ |
|  | B14 | 289 | Shufflon-specific recombinase of  *Salmonella enterica* plasmid pNF1358 | DQ017661 | 100 | 41^*^ | 9^*^ |
|  | B15 | 300 | Sc/SvP gene inversion product of *E. coli* plasmid p15B | X62121 | 99 | 12 | 1 |
|  | B21 | 209 | A hypothetical protein of plasmid pCRY of *Yersinia pestis* *biovar* Microtus strain 91001 | [AY233333](http://www.ncbi.nlm.nih.gov/entrez/viewer.fcgi?db=nucleotide&val=37927516) | 82 | 10 | 2 |
|  | B22 | 178 | A hypothetical protein of plasmid pAPEC-O1-ColBM of APEC strain O1:K1 | [DQ381420](http://www.ncbi.nlm.nih.gov/entrez/viewer.fcgi?db=nucleotide&val=88770133) | 98 | 9 | 0 |
|  | B25 | 174 | IroN of plasmid pAPEC-O1-ColBM APEC strain O1:K1 | [DQ381420](http://www.ncbi.nlm.nih.gov/entrez/viewer.fcgi?db=nucleotide&val=88770133) | 100 | 60 | 41 |
|  | B30 | 346 | Colicin B of plasmid pF166 from *E. coli* | [M16816](http://www.ncbi.nlm.nih.gov/entrez/viewer.fcgi?db=nucleotide&val=145566) | 97 | 21^*^ | 4^*^ |
|  | B32 | 510 | Transcriptional regulator and a hypothetical protein of *Yersinia pestis* Antiqua plasmid pPCP | CP000310 | 75 | 10 | 2 |
|  | B36 | 633 | Plasmid replication protein of plasmid pKL1 of *E. coli* | [U81610](http://www.ncbi.nlm.nih.gov/entrez/viewer.fcgi?db=nucleotide&val=6274515) | 99 | 29 | 14 |
|  | B39 | 209 | A hypothetical protein of plasmid pCRY of *Yersinia*  *pestis* biovar Microtus strain 91001 | [AE017044](http://www.ncbi.nlm.nih.gov/entrez/viewer.fcgi?db=nucleotide&val=45357210) | 82 | 11 | 2 |
| **Chromosome** | B1 | 328 | [Putative superinfection exclusion protein B of a prophage](http://www.ncbi.nlm.nih.gov/entrez/viewer.fcgi?val=26111730&db=Nucleotide&from=1409774&to=1410355&view=gbwithparts) of UPEC strain CFT073 | AE014075 | 99 | 11 | 29 |
|  | B2 | 251 | A hypothetical protein of UPEC strain UTI89 | CP000244 | 98 | 40^*^ | 18^*^ |
|  | B3 | 262 | Putatitive transposase subunit of PAI I_APEC-O1_ of APEC strain O1:K1 | DQ095216 | 100 | 18 | 16 |
|  | B4 | 197 | Phosphoglycerate activator (PgtA) of PAI I_APEC-O1_ of APEC strain O1:K1 | DQ095216 | 99 | 26 | 35 |
|  | B7 | 503 | [Putative Na(+)/H(+) exchanger YjcE](http://www.ncbi.nlm.nih.gov/entrez/viewer.fcgi?val=91070629&db=Nucleotide&from=4970868&to=4972541&view=gbwithparts) of UPEC strain UTI89 | CP000243 | 100 | 26^*^ | 50^*^ |
|  | B9 | 376 | A Hypothetical protein of UPEC strain CFT073 | [AE014075](http://www.ncbi.nlm.nih.gov/entrez/viewer.fcgi?db=nucleotide&val=24080789) | 85 | 4^*^ | 20^*^ |
|  | B10 | 373 | [Putative exported protein](http://www.ncbi.nlm.nih.gov/entrez/viewer.fcgi?val=91070629&db=Nucleotide&from=3125577&to=3126218&view=gbwithparts) of UPEC strain UTI89 | CP000243 | 100 | 6 | 6 |
|  | B13 | 354 | A hypothetical protein of UPEC strain UTI89 | CP000243 | 99 | 11 | 24 |
|  | B16 | 297 | A hypothetical protein of UPEC strain UTI89 | CP000243 | 100 | 3 | 15 |
|  | B17 | 228 | A hypothetical protein of UPEC strain UTI89 | CP000243 | 100 | 4^*^ | 23^*^ |
|  | B19 | 269 | [Putative protease YhbU precursor](http://www.ncbi.nlm.nih.gov/entrez/viewer.fcgi?val=26111730&db=Nucleotide&from=3735090&to=3736112&view=gbwithparts) and a hypothetical protein of UPEC strain CFT073 | AE014075 | 100 | 7 | 18 |
|  | B20 | 126 | A putative conserved protein of UPEC strain CFT073 | AE014075 | 100 | 3^*^ | 19^*^ |
|  | B23 | 376 | A hypothetical protein of UPEC strain CFT073 | AE014075 | 85 | 6 | 6 |
|  | B24 | 327 | A conserved hypothetical protein and a putative fimbrial-like adhesin protein of APEC strain O1:K1 | NC_008563 | 99 | 16^*^ | 48^*^ |
|  | B26 | 377 | A [hypothetical protein](http://www.ncbi.nlm.nih.gov/entrez/viewer.fcgi?val=26111730&db=Nucleotide&from=4327831&to=4328895&view=gbwithparts) and [transposase InsC for insertion element IS2A/D/F/H/I/K](http://www.ncbi.nlm.nih.gov/entrez/viewer.fcgi?val=26111730&db=Nucleotide&from=4329034&to=4329369&view=gbwithparts) of UPEC strain CFT073 | AE014075 | 98 | 5^*^ | 26^*^ |
|  | B27 | 201 | Putative phosphotransferase system component of genetic island GimB of APEC strain IMT 5155 | [AJ810519](http://www.ncbi.nlm.nih.gov/entrez/viewer.fcgi?db=nucleotide&val=51465230) | 100 | 10^*^ | 37^*^ |
|  | B28 | 511 | [Putative phosphotransferase system protein](http://www.ncbi.nlm.nih.gov/entrez/viewer.fcgi?val=91070629&db=Nucleotide&from=3862745&to=3863491&view=gbwithparts) of genetic island GimB of APEC strain IMT 5155 | AJ810519 | 100 | 10^*^ | 37^*^ |
|  | B29 | 213 | [Putative exclusion protein Ren of prophage](http://www.ncbi.nlm.nih.gov/entrez/viewer.fcgi?val=26111730&db=Nucleotide&from=1415485&to=1415775&view=gbwithparts) of UPEC strain UTI89 | [AE014075](http://www.ncbi.nlm.nih.gov/entrez/viewer.fcgi?db=nucleotide&val=26111730) | 100 | 17^*^ | 40^*^ |
|  | B33 | 398 | Putatitive transposase subunit of PAI I_APEC-O1_ of APEC strain O1:K1 | DQ095216 | 99 | 19 | 22 |
|  | B34 | 175 | Putative serine protease of UPEC strain UTI89 | CP000243 | 90 | 6 | 4 |
|  | B35 | 496 | A hypothetical protein of UPEC strain UTI89 | CP000243 | 100 | 15 | 20 |
|  | B37 | 217 | A h[ypothetical fimbrial adhesin YadC precursor](http://www.ncbi.nlm.nih.gov/entrez/viewer.fcgi?val=91070629&db=Nucleotide&from=155734&to=157023&view=gbwithparts) and [putative fimbrial subunit YadK precursor](http://www.ncbi.nlm.nih.gov/entrez/viewer.fcgi?val=91070629&db=Nucleotide&from=157049&to=157645&view=gbwithparts) of UPEC strain UTI89 | CP000243 | 100 | 7 | 18 |
| **Other** | B5 | 323 | Unique |  |  | 3 | 2 |
|  | B6 | 557 | Unique |  |  | 10 | 1 |
|  | B12 | 261 | Unique |  |  | 0 | 0 |
|  | B18 | 232 | Unique |  |  | 1 | 0 |
|  | B31 | 587 | Unique |  |  | 2^*^ | 17^*^ |
|  | B38 | 333 | Unique |  |  | 11 | 12 |

^A^ Note that the SFs represented only portions of individual genes or genetic elements and were by no means complete gene sequences. Some SFs represent different regions of the same gene, so have identical translated products. The SFs categorized under “other” and B2, B9, B14, B15, B21, B23, B30, B32, B36 and B39 are absent in the published databases including the APECO1:K1 genome (NC_008563).

^B^ Each category of *E. coli* contains 95 isolates. ^*^ indicates a statistically significant difference (*P*< 0.001, Fisher’s exact test, with Bonferroni adjustment).
