# Supplementary material for: Common and specific genomic sequences of avian and human extraintestinal pathogenic Escherichia coli as determined by genomic subtractive hybridization
Source: BMC Microbiol. 2007 Aug 30;7:81. doi: 10.1186/1471-2180-7-81 (PMC2031896; doi:10.1186/1471-2180-7-81)
Supplement: Additional file 4 — Summary of BLAST search results for SFs obtained as a result of SSH between UPEC 88 (tester strain) and APEC 113 (driver strain). The data provided represent the BLAST search results for SFs obtained with SSH between UPEC 88 (tester strain) and APEC 113 (driver strain), and statistical comparison of occurrence of those SFs among a collection of APEC and UPEC. [file 1471-2180-7-81-S4.doc]

**Summary of BLAST search results for SFs obtained as a result of SSH between UPEC 88 (tester strain) and**

**APEC 113 (driver strain)**

| **Location of SF** | **SF** | **Size**  **(bp)** | **Translated products of the nucleotide sequences with similarity^A^** | **GenBank accession** | **% identity** | **% of positive isolates^B^** | |
| --- | --- | --- | --- | --- | --- | --- | --- |
|  |  |  |  |  |  | **APEC** | **UPEC** |
| **Plasmid** | C4 | 367 | TnpR resolvase of plasmid pRMH760 of *Klebsiella pneumoniae* | [AY123253](http://www.ncbi.nlm.nih.gov/entrez/viewer.fcgi?db=nucleotide&val=40795472) | 100 | 14 | 22 |
|  | C8 | 521 | Truncated Tn2 transposase of plasmid pYC-14 of *E. coli* | [AY604722](http://www.ncbi.nlm.nih.gov/entrez/viewer.fcgi?db=nucleotide&val=48994625) | 99 | 17 | 17 |
|  | C10 | 188 | Transposase of plasmid pUTI89 of UPEC strain UTI89 | CP000244 | 100 | 2^*^ | 36^*^ |
|  | C11 | 135 | Resolvase of plasmid pMAR7 of *E. coli* strain E2348/69 | DQ388534 | 100 | 19 | 17 |
|  | C12 | 158 | [PilV-like protein](http://www.ncbi.nlm.nih.gov/entrez/viewer.fcgi?val=26111730&db=Nucleotide&from=2197781&to=2199244&view=gbwithparts) of UPEC strain CFT073 | AE014075 | 100 | 12^*^ | 42^*^ |
| **Chromosome** | C1 | 280 | P[utative LysR-family transcriptional regulator](http://www.ncbi.nlm.nih.gov/entrez/viewer.fcgi?val=110341805&db=Nucleotide&from=383398&to=384324&view=gbwithparts) of UPEC strain 536 | CP000247 | 100 | 53 | 69 |
|  | C2 | 344 | A hypothetical protein of *Shigella boydii* Sb227 | CP000036 | 100 | 0 | 6 |
|  | C3 | 276 | [Putative thioesterase](http://www.ncbi.nlm.nih.gov/entrez/viewer.fcgi?val=110341805&db=Nucleotide&from=2017539&to=2018261&view=gbwithparts) and a [hypothetical protein](http://www.ncbi.nlm.nih.gov/entrez/viewer.fcgi?val=110341805&db=Nucleotide&from=2018254&to=2019768&view=gbwithparts) of UPEC strain 536 | CP000247 | 100 | 2^*^ | 51^*^ |
|  | C6 | 300 | [YapH homolog](http://www.ncbi.nlm.nih.gov/entrez/viewer.fcgi?val=26111730&db=Nucleotide&from=2748603&to=2755766&view=gbwithparts) of UPEC strain CFT073 | AE014075 | 99 | 6^*^ | 28^*^ |
|  | C7 | 121 | [Putative P4-family integrase](http://www.ncbi.nlm.nih.gov/entrez/viewer.fcgi?val=26111730&db=Nucleotide&from=2194512&to=2195732&view=gbwithparts) of UPEC strain CFT073 | AE014075 | 100 | 15^*^ | 44^*^ |
|  | C9 | 191 | [Hypothetical transcriptional regulator YihW](http://www.ncbi.nlm.nih.gov/entrez/viewer.fcgi?val=110341805&db=Nucleotide&from=4269159&to=4269947&view=gbwithparts) of UPEC strain CFT073 | AE014075 | 100 | 42 | 48 |
|  | C13 | 270 | [Unknown protein encoded by ISEc8](http://www.ncbi.nlm.nih.gov/entrez/viewer.fcgi?val=26111730&db=Nucleotide&from=3497824&to=3498471&view=gbwithparts) of UPEC strain CFT073 | AE014075 | 100 | 0 | 5 |
|  | C14 | 159 | A region of PAI II of UPEC strain CFT073 | AF447814 | 100 | 14^*^ | 54^*^ |
|  | C15 | 360 | A hypothetical protein of UPEC strain CFT073 | AE014075 | 99 | 26 | 27 |
|  | C16 | 392 | [Putative arginine deiminase](http://www.ncbi.nlm.nih.gov/entrez/viewer.fcgi?val=26111730&db=Nucleotide&from=5093732&to=5094955&view=gbwithparts) of UPEC strain CFT073 | AE014075 | 100 | 25^*^ | 56^*^ |
|  | C17 | 272 | [YapH homolog](http://www.ncbi.nlm.nih.gov/entrez/viewer.fcgi?val=26111730&db=Nucleotide&from=2748603&to=2755766&view=gbwithparts) of UPEC strain CFT073 | AE014075 | 99 | 6^*^ | 28^*^ |
|  | C18 | 261 | A hypothetical protein of UPEC strain CFT073 | AE014075 | 100 | 1^*^ | 19^*^ |
|  | C19 | 166 | [YapH homolog](http://www.ncbi.nlm.nih.gov/entrez/viewer.fcgi?val=26111730&db=Nucleotide&from=2748603&to=2755766&view=gbwithparts) of UPEC strain CFT073 | AE014075 | 100 | 6^*^ | 28^*^ |
|  | C21 | 652 | *BamH*I-*Cla*I fragment that contains the uropathogenic-specific protein (Usp) of UPEC strain Z42 | AB056434 | 99 | 23^*^ | 62^*^ |
|  | C22 | 441 | Putative member of ShlA/HecA/Fha exoprotein family of and a hypothetical protein of UPEC strain CFT073 | AE014075 | 98 | 21 | 25 |
|  | C23 | 522 | Putative c-4 dicarboxylate transport binding protein and a hypothetical protein of UPEC CFT073 | [AE](http://www.ncbi.nlm.nih.gov/entrez/viewer.fcgi?db=nucleotide&val=26109707)014075 | 99 | 1^*^ | 14^*^ |
|  | C24 | 1343 | Uropathogenic-specific protein (Usp) of UPEC strain E25 | AB056435 | 100 | 5^*^ | 31^*^ |
| **Other** | C20 | 748 | Unique |  |  | 0^*^ | 17^*^ |

^A^ Note that the SFs represented only portions of individual genes or genetic elements and were by no means complete gene sequences. Some SFs represent different regions of the same gene, so have identical translated products. The SF C20 is absent in the published databases.

^B^ Each category of *E. coli* contains 95 isolates. ^*^ indicates a statistically significant difference (*P*< 0.002, Fisher’s exact test, with Bonferroni adjustment).
