# Supplementary material for: Common and specific genomic sequences of avian and human extraintestinal pathogenic Escherichia coli as determined by genomic subtractive hybridization
Source: BMC Microbiol. 2007 Aug 30;7:81. doi: 10.1186/1471-2180-7-81 (PMC2031896; doi:10.1186/1471-2180-7-81)
Supplement: Additional file 5 — Primers used in the SF prevalence study. This table shows the primer sequences that were used to PCR amplify tester-specific sequences obtained from four different SSHs. [file 1471-2180-7-81-S5.doc]

**Primers used in the SF prevalence study.**

| **Fragment Name** | **PCR primers^A^** | **Amplicon size (bp)** |
| --- | --- | --- |
| **1. SSH1** |  |  |
| A1 | ctggtcgatcaattcccagt  ggcattcatctccgaaacat | 332 |
| A2 | tcagttatgagtgcggcaag  gcgttagtcgaaaccgatgt | 527 |
| A3 | cagtcatccagaagcagcag  acgcggcagtgagtattacc | 542 |
| A4 | cgtcaacattgaccccattt  cgaccgctggtaaacactct | 457 |
| A5 | gcggctcgaatactgaaaag  ctgattcgtcgcattgaaga | 573 |
| A6 | agcaagcgcgaaaaattcta  cgatcagggcgattaacatc | 416 |
| A7 | aagttggcgatgagctgatta  caacaccaaccgcagagtta | 210 |
| A8 | accactgatacgggatccaa  cagtcatccagaagcagcag | 525 |
| A9 | cgtatttcattctggcttgatg  tcaatggctatggtgttttcag | 206 |
| A10 | gatgattggttgcgaaacag  gacagataaccaacgcaacg | 300 |
| A11 | ttaggttgccaggggattag  aaggttacacccgcagtcag | 385 |
| A12 | aatctgcctgtaaaacgagtgc  tagccatgaagggaatacctgt | 423 |
| A13 | cggagttattccattcctgaag  atcatcggcttcgtatttatgg | 331 |
| A14 | gcttcgtaaaggagtcgaagag  accgcctagctctgttttagtg | 380 |
| A15 | ctgagggggtatcaaaatctca  cgtcaatggtgtctggatca | 300 |
| A16 | atttgcatcaagaacagcagtg  gaatgattagcccgtcaggtt | 351 |
| A17 | gaaccttcgggaaactgaaag  aaaggcgaacctgtggatagt | 340 |
| A18 | acgaaggaagggacgattattt  gaatttgcctcaacttttccag | 253 |
| A19 | tcaggcgtgagatcttctacag  cctaacagcgaacatggaac | 256 |
| A20 | gtacaggaagttccgccaatag  aacactatcactgcctcaggtg | 458 |
| A21 | tgatgattgccattgagatagc  ttgcgttagtcagcacattctt | 514 |
| A22 | acgacggattacttcatcctga  cattcagattgcctttcagttg | 178 |
| A23 | gaactaaacctcaccaaagagca  tcaacgacctgagaaatcaaca | 200 |
| A25 | caagttacgacgctcaggttc  acctatcacacgagttccatga | 350 |
| A26 | tcagttttgatatggcttcgtg  catcctatacgtctgccaaaca | 238 |
| A27 | ggcgtgaactctgactttcttc  caggcatgagatcttctacagc | 199 |
| A28 | cgctgtaccaacacagctaaac  tagactaaaaacgtgccgcatc | 411 |
| A29 | agctgcagactcccattataca  atatcgctcacctcaccagatt | 237 |
| A30 | tcgttgattactgtggacctacc  cgaacatggaaccgagtagtaa | 630 |
| A31 | tattattcttggcttcggtcgt  tcgtcatcataatagccaatgc | 407 |
| A32 | tgctatcacggtaagttgttgc  cgcctgtttgatagtggatacc | 258 |
| A33 | acgcggagctttagttttagc  cgactaaaaatggatggaagga | 155 |
| A34 | tatgtatccgaacaaggtcgtc  tagttaccagccctgtcctgtt | 430 |
| A35 | gtggctatcacttcctcccata  tgatagcacccagctcagaac | 516 |
| A36 | cacaacgcgtcttcagaatg  acgagccgagatactcagga | 458 |
| A37 | gtcagaatctggctgtccctta  acgctttgcagaggactcat | 506 |
| A38 | acagctgacgcgatgatttc  cttgatgcagaggaagatggtc | 428 |
| A39 | ccgttactctgctcatacctga  ctgacaggggtgaaaaatgc | 505 |
| A40 | gtggcttttgtcgctattaacc  gtccacacaaaattcgctcata | 325 |
| A41 | cgatacgagcttgatatggatg  tgtcatcaccttcggtgtttag | 409 |
| A42 | ccatactgaaatgcgagactga  gggaaaattatctccccaaatg | 250 |
| A43 | agggtggttcaaactggcta  gcttccacaaagcacctgtaat | 176 |
| A44 | tgtcatctatgcgccttttatg  ctgatccacatcctgggtattt | 469 |
| A45 | atgctgcaagaaattgctaacg  ttgctgagtcataaacgtcctg | 370 |
| A46 | ataccgatccgctcaatcctac  gattcttcgaatggcactcc | 400 |
| A47 | cggttaaacagatgacgatcag  ctgacaatccactagcatccag | 510 |
| A48 | gcggatgtagggttgatagttc  cgtcagtcagtttacgttccaa | 304 |
| **2. SSH2** |  |  |
| U1 | aggtgcggatttcagttcac  gggtaatcccggatgagaat | 216 |
| U2 | atctcagtcgtggcaaaagg  cctactacgccatccacgat | 554 |
| U3 | ccaaaacttggagacccaac  gcggctatatgtgaggcaat | 602 |
| U4 | ccttttcgtatccagccataa  ccgtgatcattgaaagatgct | 614 |
| U5 | tgaagtggctgcaggtaaag  tttcagctcatcagcattgg | 529 |
| U6 | ccgtcttgagaagaagatcagt  cccagaaccacgtttttgat | 258 |
| U7 | tctggtgccgaaaataatcagt  tcctgtatccacctccaggtat | 527 |
| U8 | cagcgctatagctcagaaacaa  caaccgggaaaacatcactatt | 500 |
| U9 | tccctgaattcttttacgagga  ggctcttaaaggtgttggtcag | 504 |
| U10 | aggaagtgttggacttgctgat  gtaatacaatcccggtcccaaa | 150 |
| U11 | tatcttgcagtggattggtgtc  agtgaagcatgcccacaaact | 514 |
| U12 | aagatcctctgtctggtgcaat  ccgctaagatcatgccaataa | 389 |
| U13 | gggttcttgtgctgtgatgata  ccttcagatgatcgatgttcaa | 540 |
| U14 | gcagttgtggcggtaatgata  ttgtactgaatatccggaggtg | 503 |
| U15 | cacgcgtattaacagcgaagta  atagaaccgtcaaaatggctgt | 413 |
| U16 | ttttattcatggctcacagacg  ctggtcaggcgtagaaaaatct | 522 |
| U17 | gcgctacctaccgttgtcat  gtggtgcagatgaatcaacaat | 409 |
| U18 | atatctcagtcgtggcaaaagg  cttctgccagtgctcgttttt | 275 |
| U19 | ttattccgctaacacacaatgc  ccggtctgacatagtgaaagtc | 266 |
| U20 | tggagacaggagtgctaactca  attaaccgtggtgacagcagac | 329 |
| U21 | aggaggctaaaacaccctcact  cttctctggctattggcaatgt | 253 |
| U22 | gtgatcgaactggaagaaatcc  gctctacagtgaacttgctgga | 458 |
| U23 | gccatttactggaagaagatgc  gttgtaacgcactgtccacct | 429 |
| U24 | tccacaattcagcatatcgaac  ccagcagatatgtgtgggataa | 367 |
| U25 | gtacgattaacctggggaaaaa  tataaaacgcctggccggtat | 150 |
| U26 | gaacggaagataaagcgaggta  cgtgagcgttaaagatctgatg | 256 |
| U27 | accattggtaatggaggaaacc  cttctgttggctttgtgtcttg | 779 |
| U28 | caactggagcaaattgagagtg  agtaacctccagcgtgtcatct | 288 |
| **3. SSH3** |  |  |
| B1 | gatatcctgataccgaccgttt  cagcaatgagagcagaagtgat | 275 |
| B2 | tcccctttccactgattgtc  aaaagcagagtgagcctaccag | 198 |
| B3 | caggcatgagatcttctacagc  aatcggcatgaactctgacttt | 203 |
| B4 | cggagctctattactgctttgc  ttgagcatctcttttcccactt | 154 |
| B5 | ccgagcgtaccagttagaaaaa  gctggggtgatatcatcagatt | 261 |
| B6 | cgtattattctgttccgggagt  tttcattcagctaccacacgac | 452 |
| B7 | ggaagtggtatctggacaggag  ctaagtgttgacgcagaatggt | 442 |
| B8 | tgatgtttgctaccagtcgttc  gggaactgtttcagggacaag | 234 |
| B9 | tacgcgaaaagttaagggagtc  ggtagacaaggtagataaccattgc | 101 |
| B10 | aatccaaactaagctgcgtatc  gctgagatggatgctcagaaat | 353 |
| B11 | cgcctcctattttatggaaaca  tacaatctgatcacaggcggta | 187 |
| B12 | agggatggtgtaagttccgtta  atacacccgtcgttgtttctct | 215 |
| B13 | cctgtacccgatattccagaaa  tccggtgtaaaaagaacattcc | 326 |
| B14 | accggtcgtattcagtctcatt  acagaaaggcgaacgtgtcc | 271 |
| B15 | actgagctgagtgatgcctttc  accacgacacaaaaagggatag | 201 |
| B16 | gtgaagaaaacccgtgaatctc  cacttaagcgtgtcagaactgc | 225 |
| B17 | cacaaacaccagccagataaga  aagtggcgaaacgtaagtgaat | 191 |
| B18 | aacacttctgagacgggtcact  cctccgtggtgggtatataaaa | 180 |
| B19 | ccagattgaagttcccataagc  aataaaaagggcgaacacacc | 188 |
| B20 | tcagttgccaaactggatcact  caaagaagacggcaggaattt | 75 |
| B21 | caaaatggtatcgtgaacatcc  gttgaatttgccaatgagcttc | 117 |
| B22 | gaaaccggatctcagaagagaa  tgcaatttcaggctaactctgt | 127 |
| B23 | tacaggaaaggggaaatgtttg  acagccccaaatagctgtctac | 333 |
| B24 | ggaatgggatttaaaaggaagg  cgcatgtgaggaaatggtta | 294 |
| B25 | taccgcatcattctgatcgtag  cgatgttcaatgcctccttt | 152 |
| B26 | ttcatggctagctccgctac  agacttccctcctggtattgct | 353 |
| B27 | attatcgaaggtgctggaatgt  aaatacccaacacggcaataag | 178 |
| B28 | catacaggcaggcgatgaatac  ctcctgcagatgaaggtctagc | 279 |
| B29 | caggtcttctggttatcgaagg  agctcagtgatgtagatggtca | 183 |
| B30 | gtggtgtgagtgcctgttttt  tgtccccgtaatcaataccttc | 215 |
| B31 | gaaaattggcaagaaggacaag  tgctgtaggcatggagataaca | 505 |
| B32 | cagtgaaagggtcacgtcataa  ctgactgattcccattgcatc | 373 |
| B33 | cactgctttatgccaagttgag  cttccatcgagatcgggtaata | 321 |
| B34 | caaacggaacagcggtattact  attcgtttacgtggtgaagctc | 118 |
| B35 | tacacaacgtttctgagggaaa  tctcctgcgataaaatggatgt | 368 |
| B36 | aacgttagtgcatgggattttc  tatcaccgtctttgttgaccag | 478 |
| B37 | caaagtgctgcaagcattatct  tggaccaacaccgtaattattc | 165 |
| B38 | gtctgaatacacgcgaaaaatg  cgctttctctaattcgcagact | 251 |
| B39 | caaaatggtatcgtgaacatcc  gttgaatttgccaatgagcttc | 117 |
| **4. SSH4** |  |  |
| C1 | cacggtattcctgaaactcctc  aatcctcaagaatcggctgtaa | 271 |
| C2 | ccaacacatcatcctcatacca  aaaatggcggtaacactg | 339 |
| C3 | gtcgcaccggtatactatctc  gaaccacctgaatatggcaaac | 207 |
| C4 | cctgacgggcatttagtaaca  accagaatgacatcaccttcct | 338 |
| C6 | tggtaccaacagcggaaata  ccctgtccctgcaaagatatag | 207 |
| C7 | ccttacccggaagtctccttag  ctttttgctcgtgtttttcctg | 106 |
| C8 | gaagaaatggtggagcagtacg  atcaagctgatgacccagagat | 414 |
| C9 | ccgatcatacgaagtttaccg  ccgtatcaaccgaagtttcaat | 181 |
| C10 | aaagcctttatcagtgcgtcat  agtaagcgaacggagatagctg | 157 |
| C11 | gtaccgtggacaggaacgtc  ggtccctttcgtcttcaagaat | 123 |
| C12 | gctgctggttgtgaaatcactt  taatgacataatcaggcgctgt | 139 |
| C13 | gcgatcaccctactcagtacttc  cggggtaaatgttcttcaaagt | 256 |
| C14 | cgaacagcggacattcactac  atgactggatttcagaccgatt | 120 |
| C15 | ctgatggctgattttactggtg  gctcagcgttgtatgtgttagc | 328 |
| C16 | ctacgtttgctactgacgttcg  cagcacatcaccaccttctaac | 374 |
| C17 | tcgacagtaacgtctggaagaa  atcctgggtggaacttaactga | 240 |
| C18 | acagcgggcagacattaactat  cttccggtcaaaatagccatac | 212 |
| C19 | caggaaataccctgaccgtaaa  cggacattattcaccaccac | 151 |
| C20 | tcggaacaagtcttgagcagt  gcttgcttgattgttgctttc | 574 |
| C21 | tgaaacctcgtgttgtgttgt  gaaatatcgccctgcttctc | 578 |
| C22 | ggatcctatagaaccgccaat  aagatcctctgtctggtgcaa | 253 |
| C23 | tccggtaataatccctgcttt  agtgctgggagcgttaaaaat | 418 |
| C24 | ttccggacggtacagataccta  gggatttatctttgctagttgc | 653 |

^A^ Melting temperature of the primers were between 59- 61.5 °C.
